# The Role of the Sharing Economy and Artificial Intelligence in Health Care: Opportunities and Challenges

**DOI:** 10.2196/13469

**Published:** 2019-10-15

**Authors:** Huailiang Wu, Nga-Kwo Chan, Casper J P Zhang, Wai-Kit Ming

**Affiliations:** 1 International School Jinan University Guangzhou China; 2 School of Medicine Jinan University Guangzhou China; 3 School of Public Health The University of Hong Kong Hong Kong China; 4 HSBC Business School Peking University Shenzhen China

**Keywords:** health care, health care system, sharing economy, artificial intelligence

## Abstract

Health care systems worldwide have been influenced by the globally growing trend toward a sharing economy and will likely advance with these trends in the near future. Therefore, based on peer-to-peer relationships between individuals, sharing health care works by renting medical staff, facilities, and other medical resources. Medical data innovation, integration, analysis, and sharing have the potential to dramatically change the current pattern of the health care system and to provide precise and predictive medical assessment for individuals in the future. In addition, artificial intelligence could be useful in the fields of both clinical medicine and medical research and help to minimize the scarcity of human resources and broaden the role of humans in health care.

The scarcity of health care resources is a long-standing, persistent global issue that is increasing with the worldwide aging population [1]. Possible approaches toward alleviating this scarcity include applying a sharing economy model to the health care industry [2]. The concept of sharing has been incorporated into a range of commercial activities related to daily life, such as retail and transportation. The health care system has also been influenced by the globally growing trend toward a sharing economy [3] and will likely advance with these trends in the near future. Such foreseeable trends continually accompany the integration of innovative technology in the emerging big-data era, including artificial intelligence (AI). Decision making on global issues requires new technologies based on AI techniques [4]. AI techniques are also needed in clinical prediction, diagnostics, and therapeutics and can be implemented by “learning” from appropriate data (eg, in image-related specialties) [5].

China is currently experiencing rapid economic development and growing health care needs. Given these circumstances, the Chinese health care system seems ideally suited to serve as a testing ground to assess the practicality of advancing health care systems using AI.

Residents of most regions in mainland China are provided with universal health insurance, which means that patients usually only pay a small portion of the treatment fee [6]. In most circumstances, routine treatments involve visiting neighborhood clinics supported by the public sector and then hospitals, if needed. Although reform and development of the primary health care system are proceeding in China, some challenges remain including fragmented health care information and a paucity of digital data [7]. In Hong Kong, the health care system has a dual-track structure encompassing the public and private sectors [8]. Public health care, operationalized by public hospitals, is the cornerstone of the health care system in Hong Kong, accounting for approximately 90% of health services and 29% of outpatient services. Hong Kong public health care also provides a comprehensive range of quality services at very low costs (eg, US $13/bed/day), which is made possible by a high subsidy rate [7]. In Hong Kong, private clinics complement the public sector. The American system differs from those in mainland China and Hong Kong and involves a hybrid system that includes the federal government, local governments, and private funds (households and private businesses). It is estimated that American citizens have greater health care costs than other developed countries [9]; this is likely because most US health care services are delivered privately, even if they are publicly financed. Selected characteristics (physicians, hospital beds, and mean costs) of the health care systems of mainland China, Hong Kong, and the United States are compared in Table 1. The World Health Organization has specified a minimum of 2.5 health care professionals (physicians, nurses, and midwives) per 1000 people for essential primary care coverage; among the three systems detailed here, only the United States has met this criterion.

**Table 1:** Characteristics of the selected health care systems (2016).

Characteristic	Mainland China	Hong Kong Special Administrative Region	United States
Physicians per 1000 people	2.31^a^	1.90^b^	2.55^c^
Hospital beds per 1000 people	5.37^a^	5.32^b^	2.5^d^
Mean cost per patient (US $)	1801.74^a^	2208.37^b^	9990^c^

^a^Data source: National Health and Family Planning Commission of the People’s Republic of China.

^b^Data source: Department of Health, Hong Kong Special Administrative Region.

^c^Data source: US Department of Health and Human Services.

^d^Data source: 2014 World Health Organization report.

In a classical health care system, primary care is mainly provided by family physicians. Family physicians are expected to offer appropriate advice and diagnoses at the early stages of illness. This type of health care requires familiarity with patients’ conditions (eg, drug allergies or idiopathic issues) and therefore represents a patient-centered approach characterized by preventive and coordinated actions [10]. However, the corresponding costs associated with this comprehensive care tend to be high and can vary across patients. In recent years, the concept of sharing health care has emerged. Sharing health care is based on a peer-to-peer relationship between individuals with similar economic/social roles and works by renting medical staff, facilities, and other medical resources. For example, doctors will practice in single hospitals, clinics, or private health care facilities as well as other locations in a more flexible fashion. This sharing health care system will be similar to other growing sharing businesses (eg, Airbnb, Mobike, and Uber), which are characterized by low entry requirements and high efficiency [2]. In this vision of the future, family physicians will engage in medical record sharing (with informed consent) and paramedic and facility sharing; therefore, with the same limited resources, a larger proportion of patients could receive primary medical care. With the application of principles of sharing economy in medicine, more patients look for medical information on the internet and make decisions based upon that information [11].

The term crowdfunding originally refers to the collective effort of consumers who offer funding to support and sustain a project [12]. Today, an interesting application of sharing economy in health care has emerged in mainland China (eg, among users of Wechat, Weibo, QQ, and other social networking). We consider the application of sharing economy in health care as a type of “charitable crowdfunding,” which refers to crowdfunding projects that aim to help the poor, disabled, and other disadvantaged people to pay their health care budgets. As an example, since its foundation in 2015 until September 2018, one popular charitable crowdfunding project in mainland China—“fun in funding”—has already raised more than 55 billion RMB and helped 2.5 million families overcome fatal diseases such as leukemia and lung cancer. Many ordinary people donate just a few RMB, but with the spread of donation messages, more donations follow. As a result, such approaches are likely to raise the targeted money for poor and severely ill patients. Although there is no accurate or reliable statistical data of all funding used in this practice, we believe that this innovation of crowdfunding application has succeeded in helping many low-income families and people with genetic diseases to treat their illnesses. Furthermore, this can help fill the gap of the current health insurance system because all types of health insurance can only cover about 70%–80% of the expenditure if the expenditure does not exceed the reimbursement cap (Figure 1).

**Figure 1:**
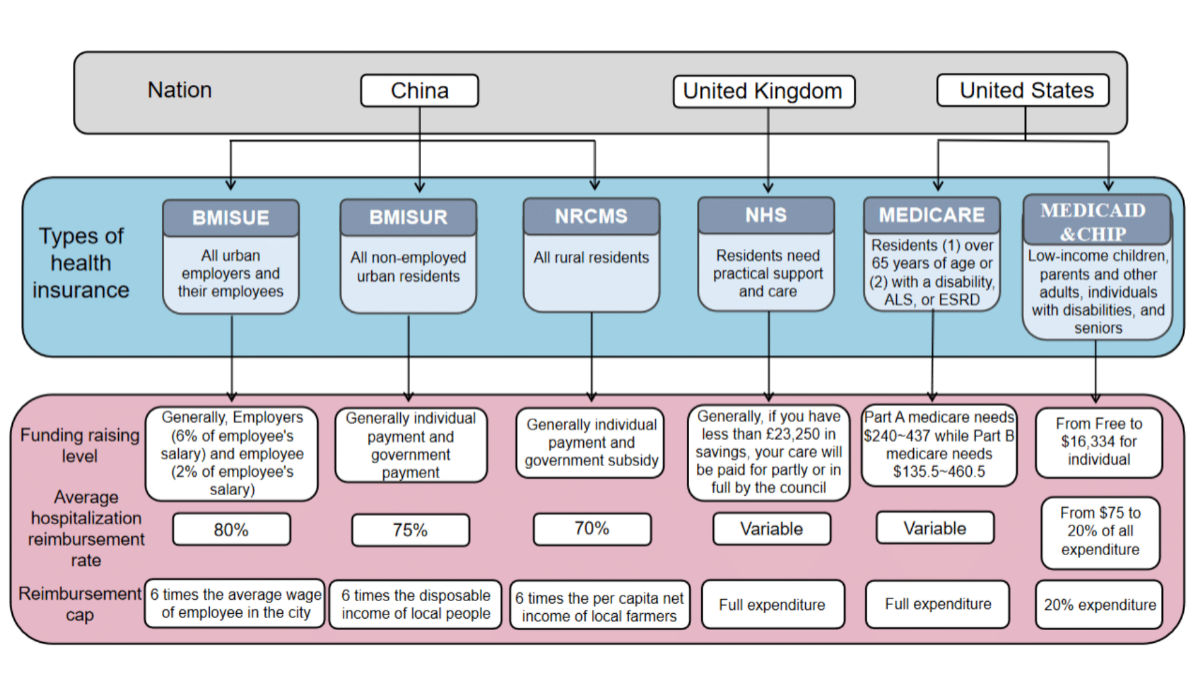
A comparison of the Health Insurance Systems of mainland China, the United Kingdom, and the United States. Source for data on BMISUE, BMISUR, and NRCMS is Han and Meng [13]. BMISUE: Basic Medical Insurance System for Urban Employees; BMISUR: Basic Medical Insurance System for Residents; NRCMS: New Rural Cooperative Medical System; NHS: National Health Service; CHIP: Children’s Health Insurance Program; ALS: amyotrophic lateral sclerosis; ESRD: end-stage renal disease.

Medical data innovation, integration, analysis, and sharing have the potential to dramatically change the current pattern of the health care system worldwide and provide precise and predictive medical assessment for individuals in the future [14,15]. In addition, decision makers should provide a sufficient medical budget and lead the digitizing health care process through cooperation among the various health care organizations (hospitals, private medical constitutes, and charitable companies) to achieve development of health care systems.

The development of AI has been considerable over the past several decades, and it is conceivable that it could be applied to the medical field, particularly to data evaluation. In about 20 years, half of all work is expected to be out of date or no longer needed, and medical health care is not exempt from AI development [16]. AI could be useful in the fields of clinical medicine and medical research and help minimize the scarcity of human resources and broaden the role of human beings in health care. Given the popularity of personal smart devices, the idea of adopting AI for personal health care services is no longer out of reach and is increasingly viable.

AI technology will indeed help health care professionals provide more care for a larger number of patients, make better clinical decisions, and reduce unnecessary hospitalization and health care costs [17]. However, it is essential to assess the ethical issues in the application of AI technology in health care. Does AI involvement change the doctor-patient relationship and who is responsible for technology-assisted decisions when patients are harmed? Besides, without the clinical physician’s involvement, how will patients be reassured and kept emotionally stable? All of these issues require further consideration if effective solutions are to be identified.

When working toward achieving these goals, governments and health care providers must obtain a deeper understanding of patients’ preferences of AI in primary care and investigate the applicability of adopting a sharing health care business model.

## Abbreviations

AI: artificial intelligence
